# An Enhanced Voxel-by-Voxel Filament Extrusion-Based Method for Realistic Radiological Phantoms: A Breast Phantom Case

**DOI:** 10.3390/polym18030395

**Published:** 2026-02-02

**Authors:** Nikiforos Okkalidis, Georgios Giakoumettis, Kristina Bliznakova, Nikolay Dukov, Zhivko Bliznakov, Georgios Plataniotis, Panagiotis Bamidis, Emmanouil Papanastasiou

**Affiliations:** 1 Medical Physics & Digital Innovation Laboratory, School of Medicine, Faculty of Health Sciences, Aristotle University of Thessaloniki, AHEPA University General Hospital of Thessaloniki, 54636 Thessaloniki, Greece; nikiforos_ok@hotmail.com (N.O.); ggiakoumettis@gmail.com (G.G.); bamidis@med.auth.gr (P.B.); empapana@auth.gr (E.P.); 2 Morphé, Lagkada 33, 54629 Thessaloniki, Greece; 3 Department of Medical Equipment, Electronic and Information Technologies in Healthcare, Medical University “Prof. Dr. Paraskev Stoyanov”–Varna, 9002 Varna, Bulgaria; ntdukov@mu-varna.bg (N.D.);; 4 Department of Radiation Oncology, School of Medicine, Faculty of Health Sciences, Aristotle University of Thessaloniki, AHEPA University General Hospital of Thessaloniki, 54636 Thessaloniki, Greece; platag@auth.gr

**Keywords:** 3D printing, voxel-by-voxel, anthropomorphic phantoms, CT imaging

## Abstract

This study introduces a novel enhanced voxel-by-voxel fused filament fabrication approach utilizing a custom 3D printer. The key innovation is the simultaneous, real-time manipulation of both filament flow and printing speed per voxel. By adjusting the printing speed proportionally to the extrusion rate, the method ensures sufficient time for precise material deposition, effectively countering under-extrusion effects and significantly improving the process’s responsiveness and accuracy. The method was validated through a calibration process and in the fabrication of a breast phantom derived from a patient’s MRI data. Calibration demonstrated a strong linear correlation between HUs, extrusion rate, and speed, with a coefficient of R = 0.99. CT scans of the phantom confirmed consistent replication of the expected HU distribution and anatomical features, visually demonstrating high correlation with the original patient images. The dual-parameter control strategy successfully enhances the fidelity of soft tissue phantoms fabrication. Future work will focus on adapting the method for high-speed printing and multi-material applications.

## 1. Introduction

Three-dimensional (3D) printing has been rapidly evolving in recent years, influencing nearly all aspects of everyday life. In particular, 3D printing has become a valuable tool across various medical fields, with significant progress made in the creation of realistic radiological phantoms [1,2,3,4,5,6,7,8,9,10,11]. The development of realistic anthropomorphic phantoms has primarily focused on three main 3D-printing approaches, including the solidification of photocurable materials and the layered deposition of melted plastic materials [1,12,13]. In several studies, multiple 3D-printing techniques have been combined, while external materials, such as muscle-equivalent liquids or bone substitutes, have also been employed to enhance realism or improve the replication of X-ray characteristics [12].

The past few years, fused filament fabrication (FFF), also known as Fused Deposition Modelling (FDM), has demonstrated remarkable advancements in printing accuracy, reproducibility, speed, and multi-material capabilities [14,15]. In the medical domain, particularly for constructing anthropomorphic phantoms, two main strategies have been mainly identified. The first involves modifying the internal structure of printed objects through variations in infill densities and patterns [16,17,18,19]. This approach is fast, easy to calibrate and well suited for radiological phantom fabrication, especially with the recent advances in high-speed, multi-material FFF/FDM 3D printing and improved repeatability. However, the process involves image segmentation, 3D modelling, and the creation of structures representing specific soft tissues with homogeneous X-ray characteristics through their volume. The second approach involves depositing melted filament either on a voxel-by-voxel basis [20,21] or within predefined local regions [22,23]. The latter method shows considerable promise for producing heterogeneous and realistic anthropomorphic phantoms using FFF/FDM 3D printing technology, as it enables direct printing from medical images. Consequently, optimal results could be achieved through the use of a single 3D printing technology, capable of accurately replicating all tissue types while also enabling the direct association of medical imaging datasets with the printing process. In recent years, such methodologies have eliminated the limitations of manual segmentation and modelling, enabling more accurate reproduction of tissue X-ray characteristics.

In this work, we present an enhanced voxel-by-voxel 3D printing method using a custom-made 3D printer for the fabrication of realistic anthropomorphic phantoms. A Magnetic Resonance Imaging (MRI) dataset was used, and the breast region was isolated and computationally converted into CT-equivalent Hounsfield Unit (HU) values, enabling the final 3D-printed phantom to replicate corresponding X-ray characteristics at a given incident X-ray beam. The results demonstrated that the replication of the breast soft tissue achieved high fidelity to the original patient data, yielding realistic outcomes in both geometry and radiological response.

The proposed method has been enhanced in comparison to the original version, providing more accurate results. While the main concept remains the same, a new parameter, i.e., the manipulation of the printing speed, has been added to improve accuracy. The novelty of this approach lies in the integration of direct voxel-based printing with an advanced extrusion technique that controls both filament extrusion rate and speed, thereby enabling the use of a single material to replicate the full range of breast soft tissues. This approach offers robust replication of X-ray imaging properties eliminating the need of multiple materials or external surrogates. Finally, the proposed method is compatible with all standard medical imaging formats and demonstrates potential for broader applications in radiology, imaging optimization, clinical training, quality assurance, and related fields.

## 2. Materials and Methods

### 2.1. Custom-Made 3D Printer with a Geared Triple-Head Extrusion System

**Figure 1:**
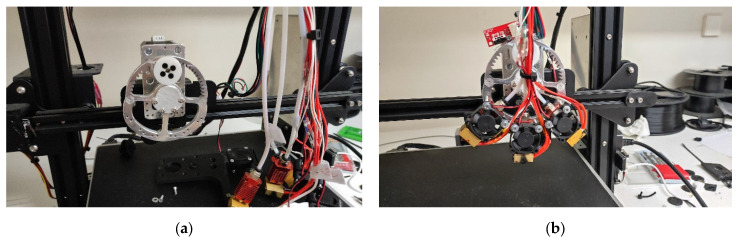
The custom-made filament extrusion-based 3D printer (**a**) with three hot ends attached on a geared system (**b**).

**Figure 2:**
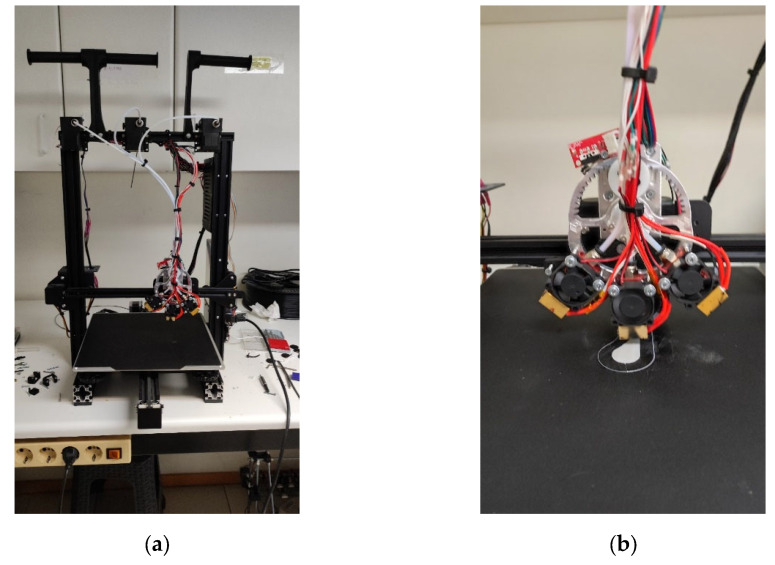
Testing the custom-made 3D printer. Full view of the printer (**a**) and during printing (**b**).

A prototype FFF/FDM 3D printer with three hot-ends attached to a geared system was developed, as shown in Figure 1 and Figure 2. An Ender-3 Max (Creality, Shenzhen, China) was used for the skeleton, and an Octopus Pro motherboard (BigTreeTech, Shenzhen, China) was installed. The geared system with a 1:4 ratio was custom-designed and fabricated using a CNC machine (Figure 1a,b). The concept of the triple-head extruder is to enable the use of different nozzles based on project requirements, enabling precise control of 3D printing accuracy in the x-y plane. Although the printer was originally designed and developed with three hot ends, only a single hot end was used in this study. Future work will explore more complex scenarios. The printing volume was 250 × 250 × 200 mm^3^, and the nozzles were 0.4 mm, 0.6 mm, and 0.8 mm, using filament with a 1.75 mm diameter (Figure 2). A custom-made software was developed in MATLAB R2022a, and the Marlin firmware was embedded on the board. The firmware was adapted, e.g., the buffer capability was increased, to prevent lag and extruder jerking caused by sending too many G-code commands per second to the board through USB or printing directly from an SD card. The specifications of the custom-made printer are presented in Table 1.

### 2.2. Enhanced Voxel-by-Voxel Method

In a conventional FDM 3D-printing process, melted filament is extruded at a fixed rate as the extruder moves across the build platform, with the slicing software determining the extrusion parameters and generating the corresponding G-code commands. The software maintains a constant ratio between the extruded filament length and the travel distance of the nozzle, while varying the extruder path to control infill density. As a result, an object’s density and weight, and consequently the HUs, are modified solely by adjusting the size of the printed area and, consequently, the amount of air gaps within the structure [16,18]. This leads to homogeneous results since each segment is produced under a specific 3D-printing pattern and infill density.

Previously, we introduced a method that replicates patient anatomy by controlling the filament extrusion rate per voxel [20]. This approach replicates HUs on a voxel-by-voxel framework, derived directly from original medical images. It was demonstrated that both soft and bone tissues could be adequately reproduced, enabling the fabrication of realistic radiological phantoms. However, the method exhibited limited performance in capturing rapid changes in pixel density, while an edge-detection algorithm was incorporated to improve imaging accuracy.

**Figure 3:**
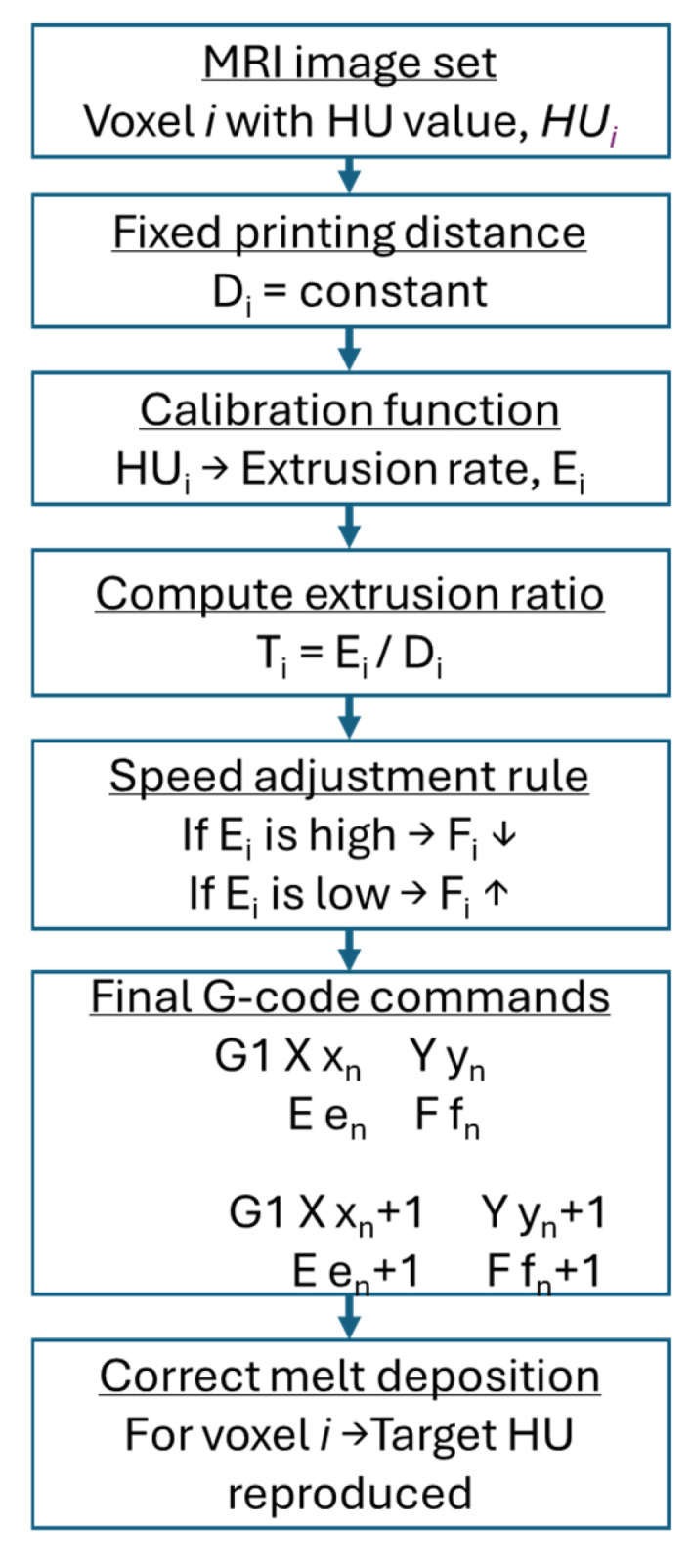
Voxel-wise conversion of Hounsfield Unit (HU) values into extrusion rate and printing speed through a calibrated G-code generation process.

In this study, manipulation of both extrusion rate and printing speed per voxel is proposed. Figure 3 presents a flowchart illustrating the proposed voxel-wise printing procedure.

Traditionally, the printing process of a line from one point to another is defined by two G-code commands [20]:

(1) $$G1\;X\;x_{n\;}Y\;y_{n\;}E\;e_{n}$$

(2) $$G1\;X\;x_{n+1\;}Y\;y_{n+1\;}E\;e_{n+1}$$

where G1 denotes a linear move of the extruder from one point to another, the values after X and Y denote the coordinates of the first and the last point in mm along the x-axis and y-axis, i.e., $x_{n},y_{n}$ are the coordinates of the starting point, and $x_{n+1},y_{n+1}$ are the coordinates of the ending point in mm. The E parameter is the extrusion rate and denotes the extruded length of filament in mm, i.e., $e_{n}$ is the first point and $e_{n+1}$ is the second point. The printing speed is commonly predefined with a single G-code command ‘G1Ff’, where F denotes the speed and f can be defined in mm/s or mm/min. Therefore, for a 3D-printed line (i) the extruded filament length ${(E}_{i})$ and the printing distance $\left(D_{i}\right)$ are calculated as follows [20]:

(3) $$E_{i}=e_{n+1}-e_{n}$$

(4) $$D_{i}=\sqrt{{\left(x_{n+1}-x_{n}\right)}^{2}+{\left(y_{n+1}-y_{n}\right)}^{2}}$$

The ratio of the extruded filament length per travelled distance $T_{i}$, is calculated as follows [20]:

(5) $$T_{i}=\frac{E_{i}}{D_{i}}$$

The current practise is to keep the parameter $\;T_{i}$ constant by adjusting both values. For example, when printing an area from 100% to 50% infill density, both $E_{i}$ and $D_{i}$ will be reduced by half, and thus $T_{i}$ will remain constant. However, the measured HUs will consequently differ between a segment printed with 100% infill density and another segment printed with 50%.

In this study, a newly enhanced method translates pixel densities of a medical image into accurate depositions of melted filament by using a series of detailed G-code commands from one coordinate point n to another coordinate point n+1:

(6) G1 X xn Y yn E en F fn

(7) $$G1\;X\;x_{n+1\;}Y\;y_{n+1\;}E\;e_{n+1\;}\;F\;f_{n+1}$$

In the proposed method, a constant *D_i_* parameter is defined in sub-millimetres, e.g., 0.4 mm or as the pixel spacing in a medical image. Then, the extrusion rate *E_i_* per voxel is calculated based on the pixel density, and thus the *T_i_* is adjusted per voxel and does not remain constant as in traditional 3D printing. Furthermore, the final amount of extruded filament is influenced by the printing speed. It has been shown that a higher printing speeds can cause under-extrusion because the selected heating temperature is insufficient to melt the filament rapidly enough [24,25]. Therefore, adjusting $F_{i}$ based on $E_{i}$ per voxel, i.e., the manipulation of the speed, affects the final amount of melted filament deposited.

When a high amount of melted filament per voxel is required, i.e., a high pixel density or HU value per voxel, the speed is reduced to allow sufficient deposition time. Conversely, when less amount of melted filament is required, the speed is increased, as less time is needed for deposition. This approach enhances the responsiveness of the 3D printing process to rapid changes in pixel density. The final correlation between HUs, extrusion rates and printing speeds is established through a calibration process, which is explained in the next sub-section.

### 2.3. Materials, HU Calibration Process, and Evaluation

**Figure 4:**
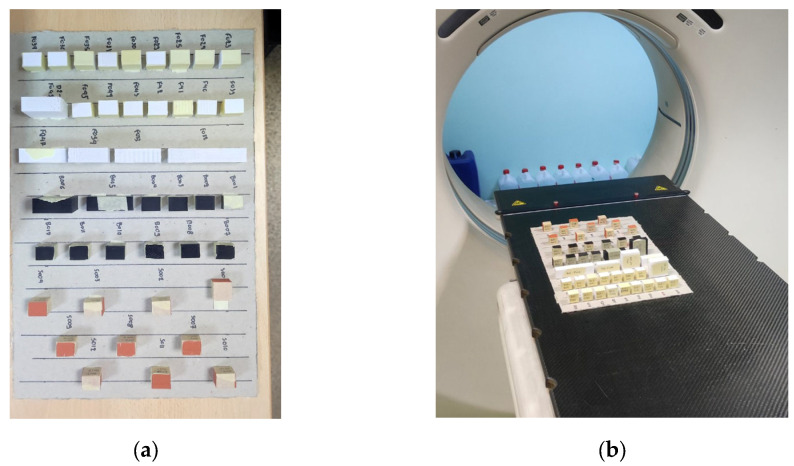
(**a**) 3D-printed calibration cuboids for testing the proposed method, placed on a CT scanner bed (**b**).

The calibration process involves 3D printing of various small cubes under different extrusion rates and speeds, followed by CT scanning those cubes to correlate the measured HUs with the applied extrusion rates and speeds. Various filament types and settings were tested during the evaluation of the proposed method, as shown in Figure 4. Figure 4a shows a top view of all the calibration cubes with their respective codes, while Figure 4b depicts the same cubes during CT scanning. For this study, i.e., for the fabrication of the breast phantom, PLA (Polylactic Acid) filament (eSUN, Shenzhen, China) with a density of 1.23 g/cm^3^ and a 0.4 mm nozzle was used for the calibration and evaluation of the proposed methodology. A layer height of 0.25 mm was chosen, and the extruder temperature was set to 180 °C, while the bed was heated to 60 °C. A series of 20 mm × 20 mm × 15 mm cuboids were 3D-printed under different extrusion rates and printing speeds ranging from 0.16 mm filament per voxel up to 0.4 mm filament per voxel and 10 mm/s to 70 mm/s, respectively. The extrusion rate in this work is defined as the extruded filament length per given voxel. Then, the cubes were CT-scanned at 80 kVp using a 2.5 mm slice thickness to measure the HUs.

**Figure 5:**
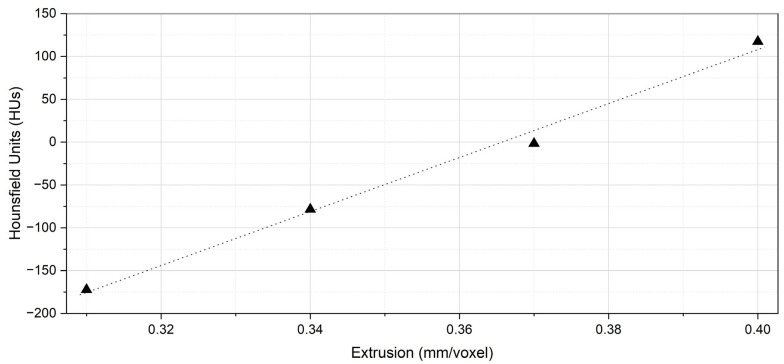
Least-squares regression model (dashed line) for predicting HUs based on the investigated parameters (triangles) from Table 2.

Table 2 shows the results of the four calibration cubes used for the correlation of the HUs with the extrusion rates and printing speeds. For the calibration process, it was decided to use the series of cuboids, with extrusion rate ranging from 0.31 mm/voxel to 0.40 mm/voxel with a 0.03 mm/voxel increments, while the printing speeds ranged from 20 mm/s to 50 mm/s in 10 mm/s step (Table 2). Four slices per cuboid were measured using a region of interest (ROI) of approximately 140 mm^2^. The mean, standard deviation (SD), maximum and minimum HU values were calculated per extrusion rate associated with the different printing speeds. A least-square line was fitted to the plot of the investigated parameters against the measured HUs, extracting the final correlation equation, as shown in Figure 5 and Equation (8). It was determined that an extrusion rate of 0.4 mm/voxel was adequate for producing a solid cube, with a high correlation observed between extrusion rate and HUs (R = 0.99). The correlation equation is as follows:

(8) HUs=3151.8E−1152.6

**Figure 6:**
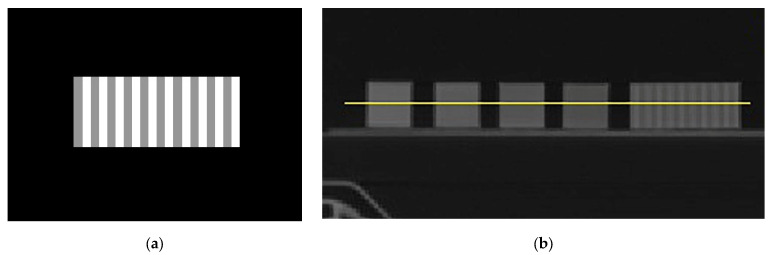
(**a**) The test sample with 2 mm-wide stripes corresponds to 0.31 mm/voxel at 50 mm/s (grey colour) and 0.37 mm/voxel at 30 mm/s (white colour). (**b**) Profile line (yellow line) drawn across the calibration cubes and the test sample for measuring the Hounsfield Units (HUs).

**Figure 7:**
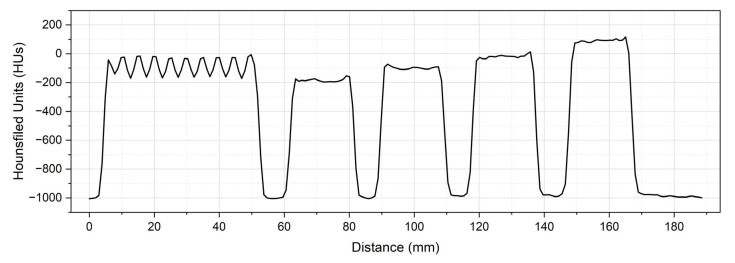
Hounsfield Units (HUs) results from the profile plot drawn across the calibration cubes and the test sample (Figure 6b).

To further assess the response of the proposed method, a test sample was 3D-printed with 2 mm-wide stripes using two parameter configurations selected from Table 2. These configurations corresponded to 0.31 mm/voxel at 50 mm/s (grey colour) and 0.37 mm/voxel at 30 mm/s (white colour), as shown in Figure 6a. Figure 6b illustrates a profile line drawn across the four calibration cubes and the test sample containing the different HU stripes. Figure 7 presents the resulting profile plot from the four cuboids, as shown in Table 2, labelled as ‘Cube 1’, ‘Cube 2’, ‘Cube 3’, and ‘Cube 4’, and the corresponding test sample shown in Figure 6a, labelled as ‘test sample’. The test sample demonstrated a high correlation between the selected configurations and the corresponding HUs.

Three-dimensional breast imaging datasets are available from CT (helical breast CT and cone-beam CT) and MRI imaging modalities. Although breast CT techniques exist, they are not widely distributed, and researchers working in this field lack easy access to the necessary infrastructure. A more accessible alternative for obtaining realistic and anatomically accurate breast data is contrast-enhanced breast MRI [26], as used in our recent studies [27]. This imaging modality has great potential due to its high sensitivity and specificity. Importantly, MRI technology is widely adopted, making the resulting datasets much more readily available. Furthermore, the use of contrast agents yields high-contrast images that allow for clear distinction between images of adipose, glandular, and skin tissues.

**Figure 8:**
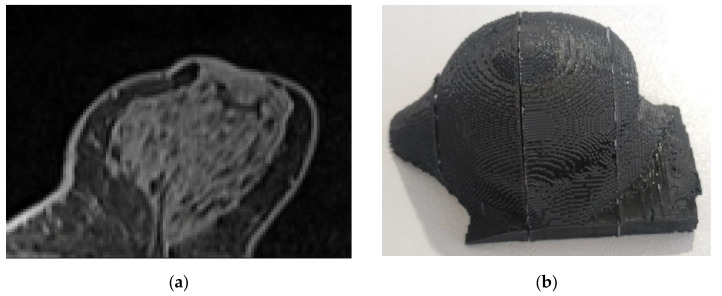
(**a**) Original MRI image in ‘.tif’ format (window 258/levelling 170). (**b**) The produced phantom.

The use of the employed data for this study was approved by the Ethics Committee of Medical University of Varna (Approval number 102/22.04.2021). An MRI dataset was acquired from a female patient using a Siemens Verio 3T scanner. Following the image acquisition, the region of interest corresponding to the breast area was segmented. The resulting images were exported in ‘.tif’ format. The final processed dataset featured pixel intensity values scaled to an 8 bit greyscale depth (0 to 255 range), with an isotropic voxel resolution of 0.8 × 0.8 × 0.8 mm^3^. An image slice from this image volume is shown in Figure 8a. Then, a grey value range of 50 to 255 was assigned to the HU range of −100 HU to 100 HU. This mapping was based on the assumption that the maximum grey value (255) in the patient’s images corresponds to skin, which typically exhibits the highest HU values in this anatomical region (approximately 100 HU). Conversely, the lowest values are expected from adipose tissue, leading to the assignment of the lowest grey value (50) to −100 HU. It was assumed that the range from −100 HU to 100 HU corresponds to the typical X-ray characteristics of human breast soft tissues.

Then, Equation (8) was used to correlate the HUs with the corresponding extrusion rates and printing speeds, and the final result of the produced phantom is shown in Figure 8b. Consisting of three distinct sections, the phantom was assembled and oriented such that its 3D-printing layers were perpendicular to the CT axial scan acquisition.

## 3. Results

**Figure 9:**
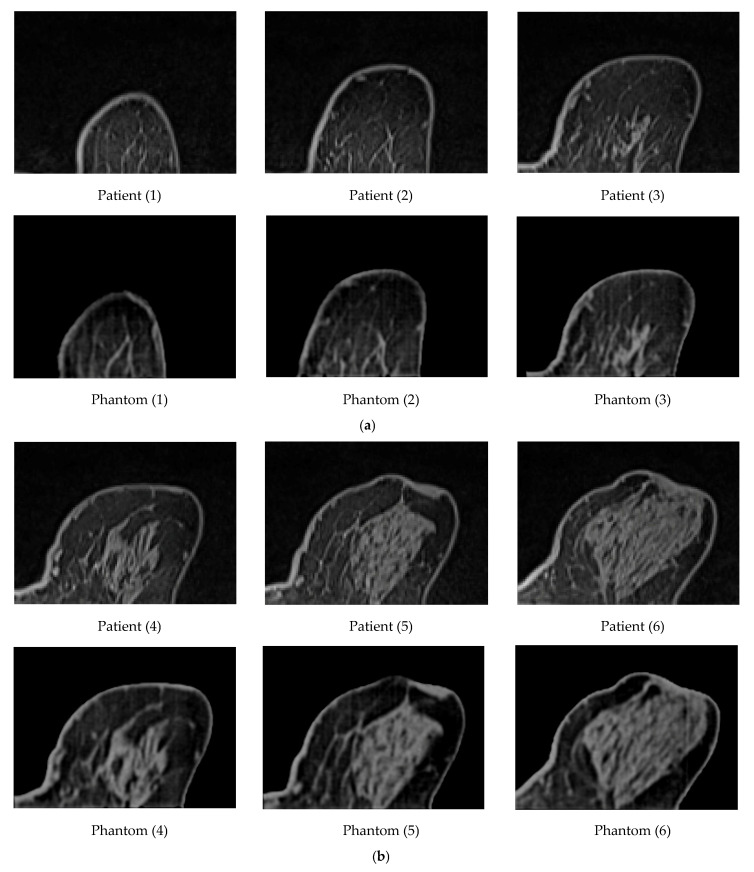

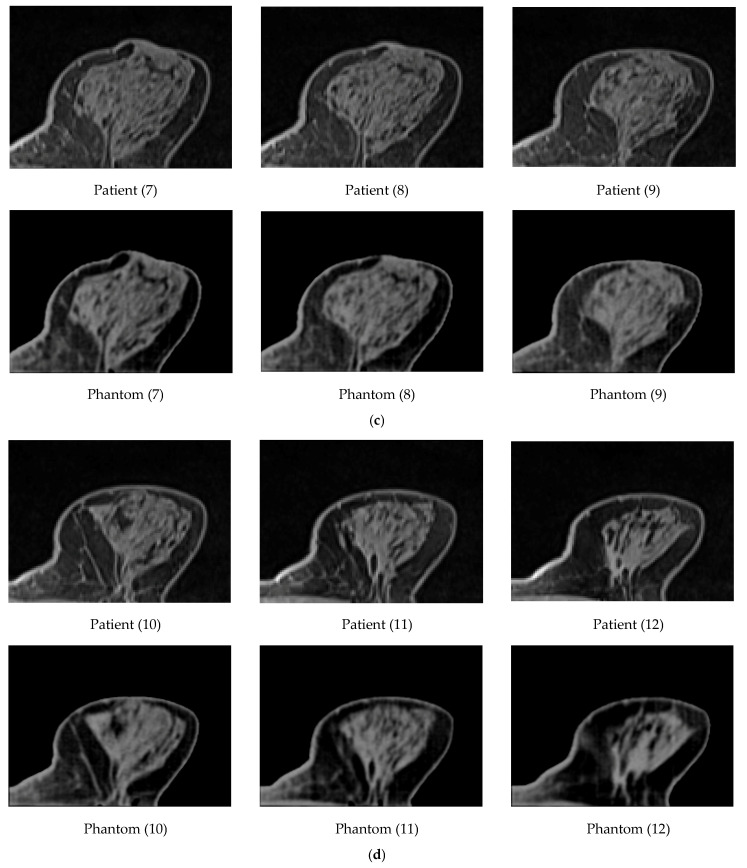
Twelve (12) images (first raw in (**a**–**d**)) of the patient and twelve (12) images from the phantom (second raw in (**a**–**d**)) were selected for comparison.

A GE Lightspeed 4-slice CT simulator was used for the CT scanning of the produced phantom. Twelve (12) original images of the patient and twelve (12) from the phantom were selected for comparison, as shown in Figure 9. Specifically, this figure presents representative axial slices from patient data and the corresponding 3D breast phantom. Each slice contains realistic distributions of glandular and fatty tissues. At the level of the original images, no visually striking differences are expected, as the phantom is designed to reproduce the anatomical appearance and attenuation properties of real breast tissue. Therefore, the similarity between patient and phantom images demonstrates the anatomical realism of the phantom rather than highlighting distinct visual features.

**Figure 10:**
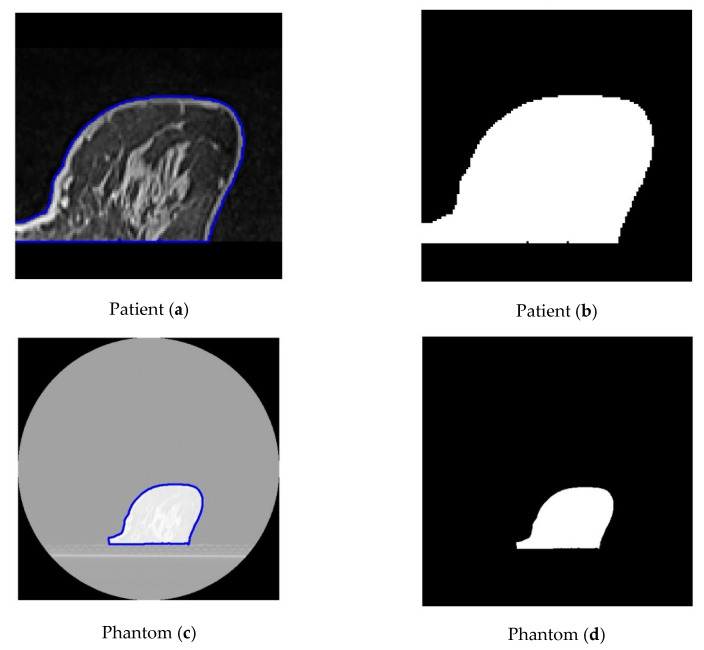
The breast area was isolated (blue contour line) using a threshold technique for both the patient’s (**a**,**b**) and the phantom’s (**c**,**d**) images; then, histograms were calculated for each segmented area.

**Figure 11:**
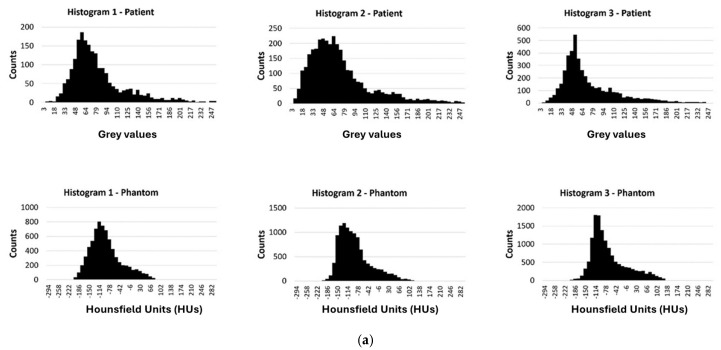

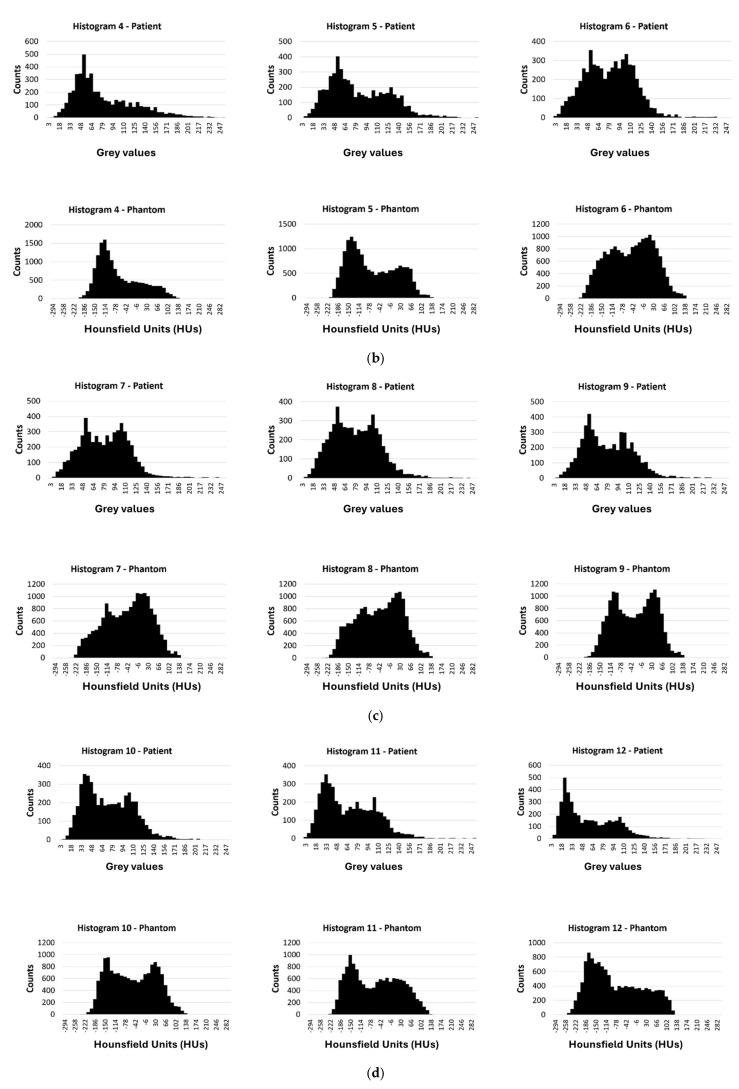
Histograms of the breast area isolated using a threshold technique for both the patient’s and the phantom’s images from Figure 9. Twelve (12) histograms of the patient images (first raw in (**a**–**d**)) and twelve (12) histograms of the phantom images (second raw in (**a**–**d**)).

**Figure 12:**
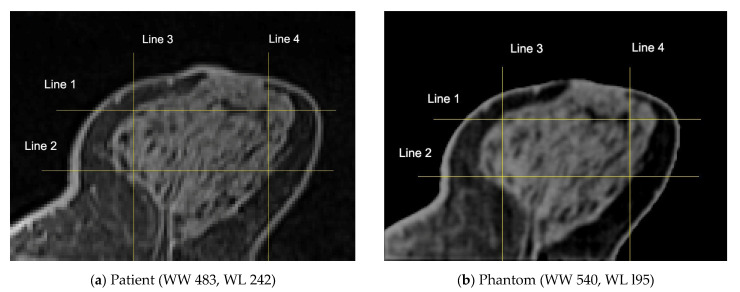
Four profile lines (yellow lines) drawn roughly at the same positions for both (**a**) patient and (**b**) phantom images.

**Figure 13:**
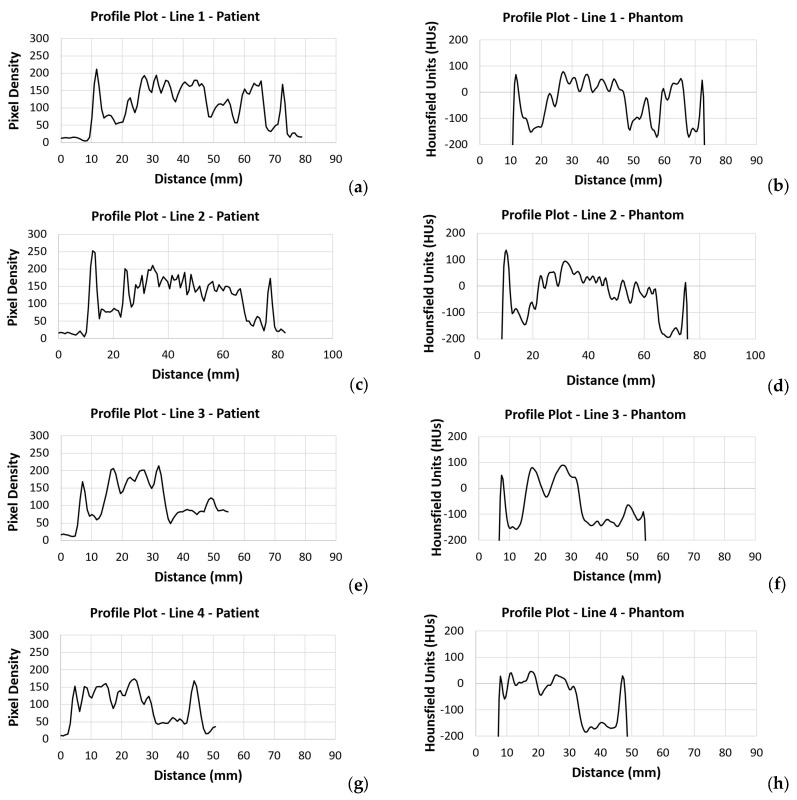
Results from four (4) profile plots drawn as shown in Figure 12 for the patient (**a**,**c**,**e**,**g**) and for the phantom (**b**,**d**,**f**,**h**) images.

To optimize visualization, a window width (WW) of 483 and a window level (centre, WL) of 242 were applied to the original patient images. For the phantom data, a WW of 540 and a WL of 95 were selected, respectively. The results led to a high correlation between the patient’s images and the phantom’s images. The breast area was isolated using a threshold technique for both the patient’s and the phantom’s images, as shown in Figure 10. The histogram of each image (Figure 11) was calculated, and showed a similar pattern, demonstrating that the method was sufficiently accurate to produce HUs consistent with those defined by Equation (8) and used for the fabrication of the phantom. It can be observed that the diagram patterns follow the initial calibration process, where a grey value of 50 corresponds to −100 HU (adipose tissue), while a grey value of 255 corresponds to 100 HU (assigned to skin and gland), across all compared images. Finally, four (4) plot profiles were drawn in similar positions, once again demonstrating a pattern consistent with the produced phantom, as shown in Figure 12 and Figure 13.

Generally, it can be noticed that the original patient images are sharper than the phantom images (Figure 9), and, upon closer observation, some minor tissue components may be missing. This can also be seen in Figure 13, where profile plots were drawn at approximately similar positions in both the patient and phantom images. It should be noted that the phantom images were selected manually in an attempt to identify the most representative slices compared to the original patient images; therefore, the images are inherently affected by contributions from the CT slices located above or below the selected slice. The use of a narrower nozzle, or a CT scanner with lower pixel spacing, could potentially improve the sharpness of the phantom images.

## 4. Discussion

In this study, a new enhanced voxel-by-voxel 3D-printing approach was developed and evaluated using a custom-made FFF/FDM 3D printer. The method introduces a simultaneous manipulation of both filament flow and printing speed per voxel, instead of maintaining a constant extrusion rate as in conventional methods. By adjusting the printing speed proportionally to the extrusion rate, the proposed technique enables accurate control of the deposited filament amount and improves the responsiveness of the process when rapid changes in pixel density occur. The method was validated through a calibration process and further by fabricating a breast phantom derived from an MRI data set. The resulting phantom demonstrated a high correlation between the printed and the original patient images, confirming that the proposed approach can successfully reproduce the anatomical features from medical images.

In our previous voxel-by-voxel method [20], the replication of medical images was achieved solely through variation in the filament extrusion rate. While this strategy enabled voxel-level control of material deposition, it demonstrated limited performance when reproducing regions with rapid spatial variations in pixel density. To partially address this limitation, an edge-detection algorithm was incorporated to enhance boundary representation and improve local image fidelity. Nevertheless, relying solely on the extrusion rate limited the responsiveness of the printing process, particularly in areas requiring abrupt changes in deposited material volume.

The present approach adds an additional degree of control by manipulating both extrusion flow and printing speed in real time. This dual-parameter control enhances the material’s physical response, and mitigates under-extrusion effects, by providing sufficient time to accurately deposit the calculated melted amounts of filaments per voxel [24,25]. Specifically, higher extrusion demands, corresponding to voxels with higher pixel HU values, are paired with reduced printing speeds, allowing adequate time for filament melting and precise material deposition. Conversely, lower extrusion requirements are combined with increased printing speeds, as less deposition time is required. This adaptive coupling enables consistent melting behaviour of the filament throughout the entire printing process, even in regions with rapidly changing of density values.

An interesting approach was presented by May et al. [23] by implementing a local area deposition strategy based on a region equal to four times the nozzle diameter. In their method, the extrusion rate was maintained as a constant parameter, and material deposition was instead regulated through variations in printing speed. This strategy exploits the under-extrusion effect to achieve local density modulation. In contrast, the method proposed in the present study avoids reliance on under-extrusion as a control mechanism and instead seeks to maintain stable extrusion conditions while dynamically adapting both flow and speed to accurately reproduce voxel-level image information.

Overall, the correspondence between the measured Hounsfield Unit (HU) values and the established calibration model remained satisfactory, indicating stable and reproducible performance of the proposed approach. In addition, the strong visual similarity observed between the original patient images and the corresponding CT scans of the printed phantom further supports the robustness of the method, as illustrated in Figure 9. Both quantitative HU agreement and qualitative image resemblance suggest that the proposed voxel-wise control strategy is capable of reliably reproducing clinically relevant density patterns.

With respect to the anatomical realism and HU replication accuracy, a single material was used similar to a study using our previous technology [27], in which a segmentation-based approach was used to convert MRI data into a radiological phantom. The use of MRI data provided an excellent foundation for anatomical reproduction, as MRI offers high soft tissue contrast compared to CT. This advantage enables clearer differentiation between glandular and adipose tissues, which is essential for generating anatomically realistic breast phantoms. Consequently, selecting MRI as the base imaging modality allowed the proposed method to overcome a key limitation of traditional CT-based approaches, which often struggle to capture subtle density variations within soft tissue structures. Despite these advantages, certain limitations persist, particularly regarding the direct translation of MRI pixel intensities into corresponding HU values. While the calibration process enables voxel-wise density control, MRI signal intensities do not have an inherent quantitative relationship with HUs, which can limit absolute HU accuracy in complex heterogeneous regions. To address this challenge, a hybrid modelling strategy could be adopted in future work. Such an approach would combine segmentation-based tissue classification, distinguishing between glandular, adipose, and skin tissues, with voxel-wise modulation of extrusion and printing parameters. This hybrid model is expected to enhance glandular tissue replication by preserving local texture while maintaining the HU correlation accuracy obtained through the calibration process.

The results from the new proposed method shown a strong correlation between HUs, extrusion rate, and speed, with R = 0.99 obtained during the calibration stage. This high correlation indicates that the combined manipulation of extrusion flow and printing speed enables accurate control of material deposition in accordance with the target image density. Histogram analysis of the CT-scanned phantom further confirmed the effectiveness of the proposed reproduction approach. The measured intensity distributions closely matched the expected HU profiles across the investigated regions, indicating a consistent and reliable translation of digital image information into physical material properties. Specifically, a linear relationship was observed between grey-level intensity values and HU values, where a grey value of 50 corresponded to approximately −100 HU, while a grey value of 255 corresponded to 100 HU, as illustrated in Figure 11. The resulting histograms were highly representative of the intended density reproduction, supporting the validity of the calibration process.

It should be noted that a limited number of measured HU values fell outside the defined calibration range. This behavior was expected, as similar density extremes were present in the original patient imaging data used as the input. Moreover, the evaluation was based on volumetric CT data rather than single-slice measurements. Consequently, the reported HU values inherently reflect contributions from adjacent slices located above and below the region of interest. Such volumetric averaging effects introduce local variations in measured density due to partial volume effects. However, given that the primary objective of the study was the development of a realistic radiological phantom rather than an idealized, noise-free representation, these fluctuations are considered acceptable and physiologically relevant. The observed variability therefore reflects realistic imaging conditions and further supports the suitability of the proposed method for medical imaging applications.

The versatility of this novel 3D printing technology extends beyond the PLA material used, since the same condition may easily be applied. The method has been already tested with Polypropylene, Acrylonitrile Butadiene Styrene (ABS), and Stonefil (PLA + Gravitational stone), showing that it could be applicable to a wide range of commercially available polymers, even including commodity plastics such as engineering-grade materials like Nylon and Polycarbonate, and high-performance systems such as Polyether Ether Ketone.

An important limitation of the proposed approach is the high printing time, especially at a time when most FDM 3D printing technologies are focused on achieving high-speed printing. Thus, our primary focus for the future is to further develop the proposed approach so that it can be executed under high-speed printing conditions. Finally, we will investigate the use of the proposed method in a multi-material setup. The incorporation of multiple printable materials with distinct radiological properties would enable more realistic replication of heterogeneous tissue types, including both soft tissues and bone. Such a development would further enhance the clinical relevance of the produced phantoms and expand the applicability of the method to a broader range of medical imaging and dosimetric studies.

## 5. Conclusions

A 3D printing method was successfully developed and tested to produce anthropomorphic radiological phantom based on the integration of direct voxel-based printing with an advanced extrusion technique that controls both filament extrusion rate and speed. The proposed methodology will increase the repeatability of the 3D-printing process for the fabrication of realistic phantoms, demonstrating a high response to significant changes in the estimated amounts of melted filament.

## Figures and Tables

**Table 1:** Specifications of the custom-made 3D printer.

**Component**	**Description**
Skeleton	Ender-3 Max
Motherboard	Octopus Pro 1.1v
Build Volume (x-y-z)	250 × 250 × 200 mm^3^
Nozzle diameter (triple-head extruder)	0.4 mm, 0.6 mm, 0.8 mm
Firmware	Marlin

**Table 2:** Mean, SD, maximum, minimum measured HUs of the calibration cubes for four slices using 80 kVp and 2.5 mm slice thickness under different extrusion rates and speeds.

Label	Extrusion Rate (mm/voxel)	Printing Speed (mm/s)	Mean HU	SD HU	Max HU	Min HU
Cube 1	0.40	20	117	27	96	143
Cube 2	0.37	30	−2	19	−37	30
Cube 3	0.34	40	−78	22	−107	−41
Cube 4	0.31	50	−172	12	−201	−125

## Data Availability

Dataset used in this article can be found at https://doi.org/10.5281/zenodo.17904503.

## Abbreviations

The following abbreviations are used in this manuscript:

FFF	Fused Filament Fabrication
FDM	Fused Deposition Modelling
PLA	Polylactic Acid
ABS	Acrylonitrile Butadiene Styrene
MRI	Magnetic resonance Imaging
HU	Hounsfield Unit
CT	Computed Tomography
3D	Three-dimensional
